# Impact of Ireland’s new diagnostic imaging referral pathway: a gp-based cross-sectional analysis

**DOI:** 10.1007/s11845-025-04119-8

**Published:** 2025-11-08

**Authors:** Fintan Stanley, Mike O’Callaghan, Ronan Fawsitt, Geoff McCombe, John Broughan, Claire Collins, Diarmuid Quinlan, Walter Cullen

**Affiliations:** 1 Irish College of GPs (ICGP), Dublin, Ireland; 2 Ireland East Hospital Group (IEHG) GP Research Network, University College Dublin/Ireland, Dublin, Ireland; 3 School of Medicine, University College Dublin (UCD), Dublin, Ireland

**Keywords:** Diagnostic Imaging, Family Practice, General Practice, Health Policy, Health Services Accessibility, Primary Health Care

## Abstract

**Background:**

General practitioners (GPs) in the Republic of Ireland have historically faced limited access to diagnostic imaging. A new national initiative allowing GPs to refer patients to private providers for imaging studies may alter referral patterns and improve patient care.

**Aims:**

This exploratory study aimed to evaluate the perceived impact of expanded GP access to diagnostic imaging on referral patterns and patient care quality in Irish primary care.

**Methods:**

A cross-sectional analysis was conducted of 620 diagnostic imaging referrals from 45 GP clinics across all Community Health Organization (CHO) regions, spanning June 2021 to August 2022. Data included imaging modalities (X-ray, DEXA, CT, MRI), referral frequencies to emergency departments (EDs), acute medical units (AMUs), outpatient clinics, and GP perceptions of care quality. GPs also provided retrospective assessments of how they would have managed each case prior to the initiative.

**Results:**

ED and AMU referrals decreased by 89%, while outpatient clinic referrals declined by 53%. Concurrently, management of cases within GP settings rose substantially. In total, 91% of participating GPs reported improved patient care quality, citing more timely and accessible imaging as a key factor.

**Conclusion:**

Despite the study’s exploratory design and reliance on retrospective GP assessments, these findings suggest that broadening GP access to diagnostic imaging may reduce hospital-based referrals and enhance patient care. Ongoing monitoring and further research are recommended to confirm the long-term impacts and sustainability of this initiative.

## Introduction

Healthcare systems globally are prioritizing early intervention in primary care as a more equitable, efficient, and economical approach [1]. This principle underpins the Republic of Ireland’s (ROI) “Sláintecare” plan, which aims to shift a substantial portion of care for Ireland’s five million citizens from secondary to primary care settings [2].

Diagnostic imaging, crucial for early disease detection and treatment planning, has historically been limited for general practice patients in Ireland, especially for advanced modalities such as computed tomography (CT) and magnetic resonance imaging (MRI) [3–5]. However, since late 2020, a new Health Service Executive (HSE) pathway has allowed general practitioners (GPs) to refer patients for various imaging studies through private providers, with public funding ensuring accessibility regardless of patients’ financial status [6, 7]. The scheme is referred to here as “GP Diagnostics”. This initiative facilitated over 250,000 imaging studies in 2022, suggesting a significant impact on healthcare delivery [8].

A recent scoping review by Phelan et al. explored GP access to diagnostic imaging across multiple healthcare systems (2012–2022), highlighting potential benefits, including reduced hospital referrals and admissions, enhanced patient care, and improved disease outcomes [9]. The review also noted the feasibility and cost-effectiveness of direct access interventions.

In Ireland specifically, O’Riordan et al. found that increased access to diagnostics could potentially reduce hospital admissions [5]. Similarly, Hughes et al. demonstrated that direct access to ultrasound for GPs yielded comparable rates of positive diagnostic outcomes relative to hospital outpatient referrals [10]. These findings align with international data, which generally indicate improved patient satisfaction and reduced waiting times in primary care settings.

The Irish health system is currently undergoing reorganization with the establishment of nine Community Healthcare Organisations (CHOs), designed to improve local service access and enhance community healthcare management. This restructuring provides the backdrop for the implementation of the new GP Diagnostics referral pathway. While X-ray and DEXA scans were broadly available prior to the initiative, the COVID-19 pandemic highlighted the need for more robust community-based diagnostic services. GP Diagnostics, part of a broader strategy to reduce hospital attendance, thus became particularly relevant, expanding GP access to imaging modalities including X-ray and DEXA [11].

Despite the significant uptake of the GP Diagnostics pathway, its formal evaluation remains limited. This study aims to address that gap by assessing the perceived impact of the GP Diagnostics referral system from the GP perspective, focusing on effects on patient management, referral patterns, and the broader health service. By exploring these impacts, we can inform future policy decisions and potentially improve the efficiency and effectiveness of primary care services in Ireland and similar health systems worldwide.

## Method

### Setting

GPs across Ireland were invited to participate in this study between September 2022 and March 2023 via GP and Irish College of General Practitioners (ICGP) networks, including webinars and working groups. Interested GPs received a study information leaflet and a consent form. Inclusion criteria were:

1. Providing a signed consent form and a HSE secure email address,
2. Having actively used the GP Diagnostics pathway during the study period (June 2021 to August 2022).

A HSE-funded research grant was offered to participating GPs (270€).

### Ethics

Ethical approval was granted by the ICGP Research Ethics Committee on 28 October 2021 (ICGP REC 210046). All patient data were anonymized at the source before being shared with the research team. Participating GPs only provided anonymized referral information, ensuring full patient confidentiality.

### Sample size and statistics

Power calculations were performed to guide sample size. It was hypothesized that the GP Diagnostics would increase the proportion of cases managed in primary care from 10 to 30%. Using a type I error (α) of 0.05 and a power (1 − β) of 0.8, a standardized difference of 0.28 was derived, indicating a target sample of 400–600 cases. While not a strict requirement—given that this was not a randomized trial with a definitive control group—this range was considered sufficient for detecting meaningful changes in referral patterns. Geographic representation and the clustering of cases within each GP practice were also considered.

**Fig. 1:**
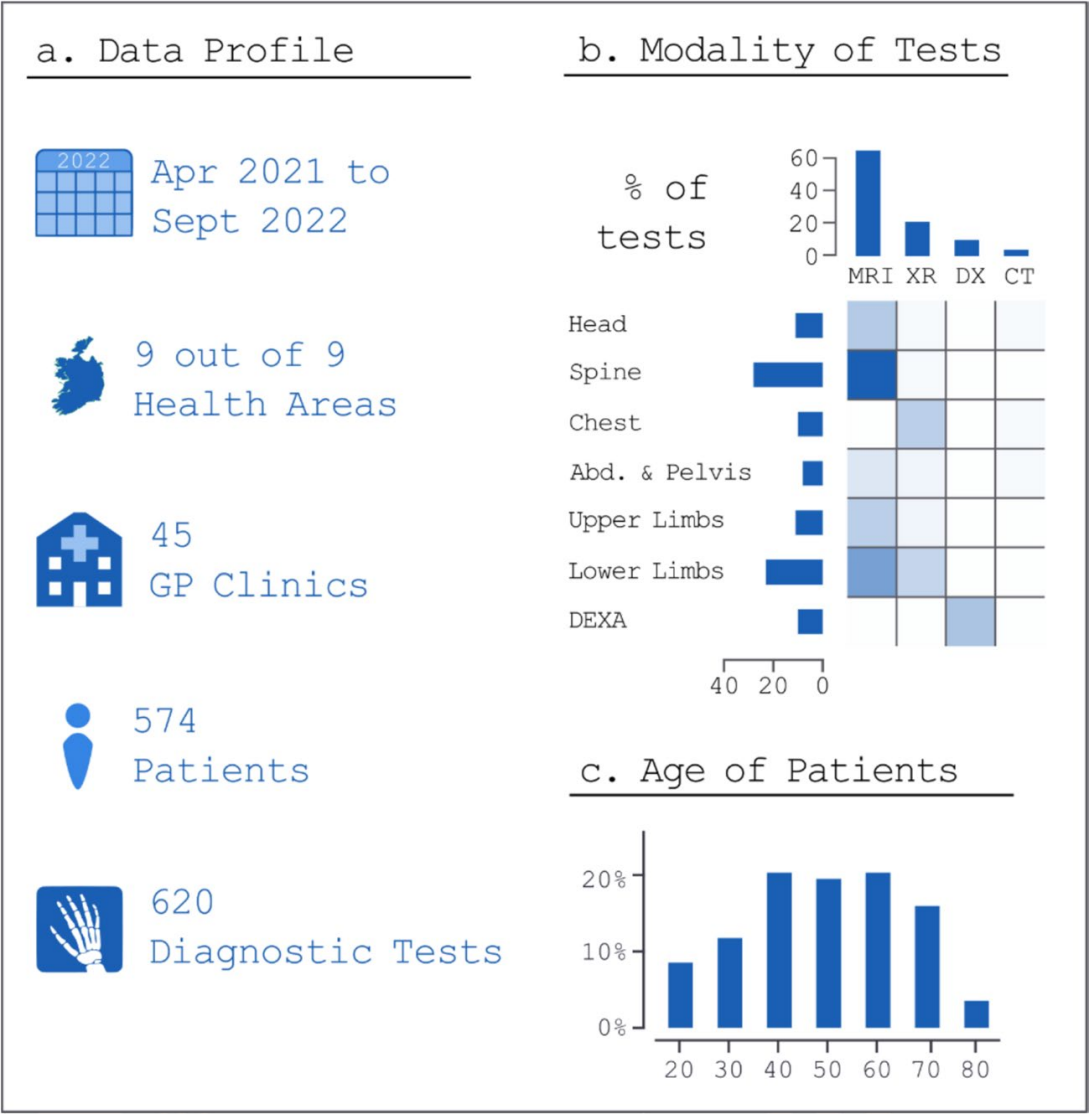
Breakdown of imaging studies by number, modality and patient age (n = 620)

Changes in referral proportions (to acute, outpatient, or primary care settings) were analysed using exact McNemar’s tests, with Bonferroni corrections applied for multiple comparisons. All statistical analyses were performed using R (R Foundation for Statistical Computing, Vienna, Austria) (Fig 1).

### Data collection

Data were collected in collaboration with private imaging providers, whose administrative teams extracted referral records from June 2021 to August 2022. Up to 15 referrals per GP were randomly selected, aiming for balanced geographic distribution. For each referral, the originating GP conducted a retrospective chart review and completed five standardized questions (see Appendix 1). These questions included how they *actually managed* the patient under the new pathway, and how they *hypothetically would have managed* the same patient under the prior system. As such, the “pre-pathway” data are based on the GP’s judgment of likely referral decisions in the absence of direct imaging access. All data were anonymized before analysis, and data management was carried out using R and QlikView [12, 13].

### Assessment of referral patterns

**Fig. 2:**
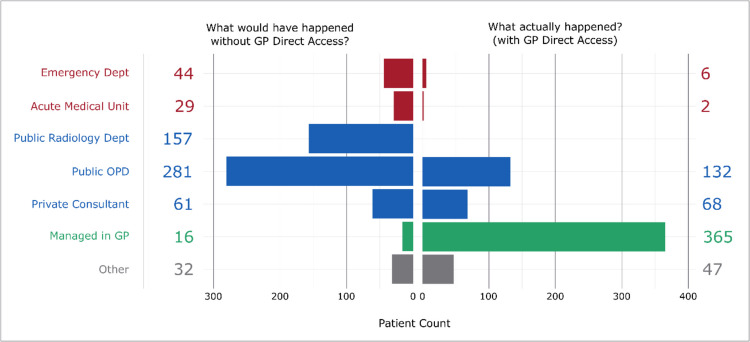
Breakdown of imaging studies by GP reporting of case management with or without the new referral pathway (n = 620)

**Fig. 3:**
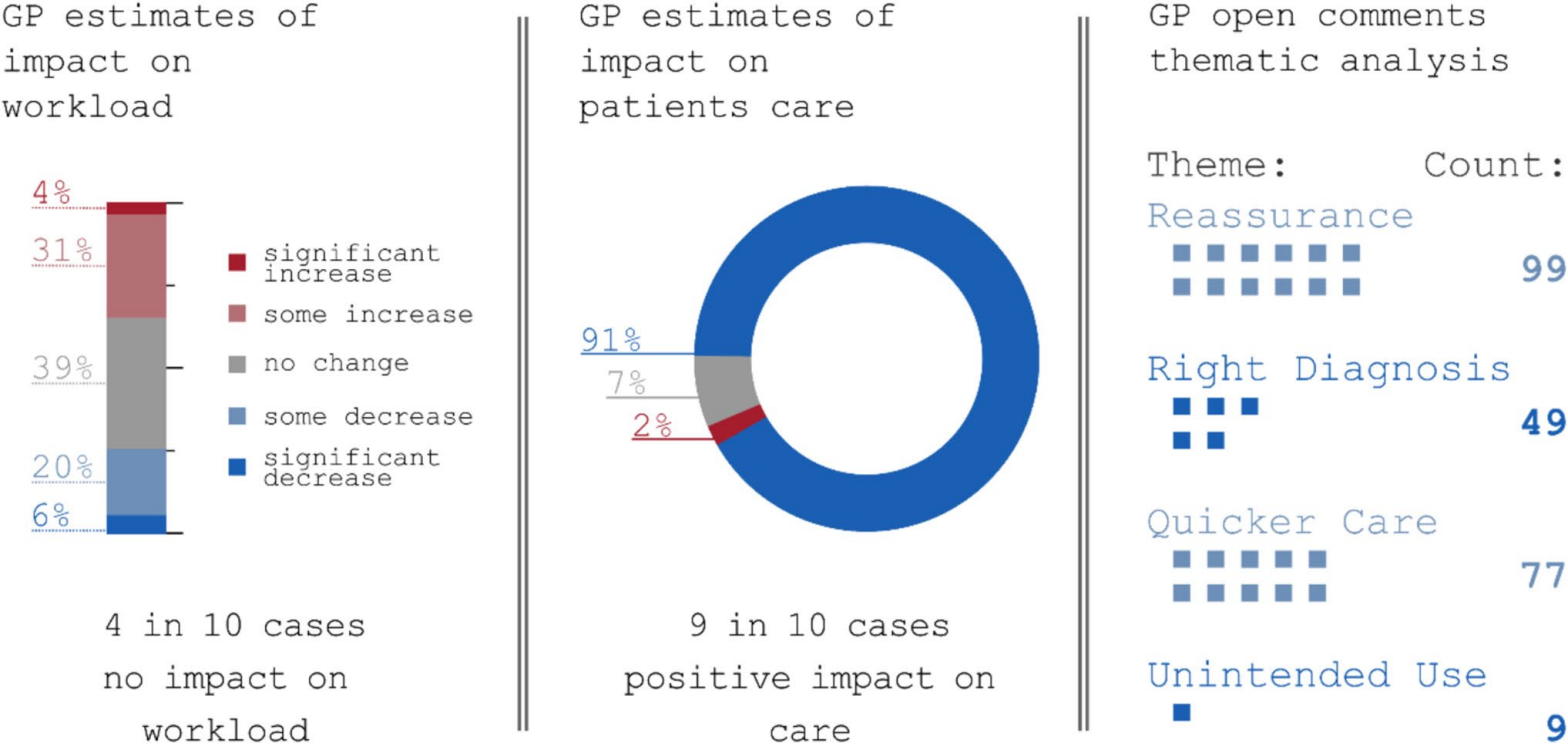
Breakdown of imaging studies by GP reported (**a**) impact on workload and (**b**) patient care (n = 620). Highlights of main emergent topics from free text GP comments (n = 338)

To evaluate the GP Diagnostics pathway’s impact, referral patterns under the existing system (direct access to MRI, CT, DEXA, X-ray via private providers) were compared with hypothetical referral patterns under the older system (Fig. 2). Previously, GPs who identified a need for diagnostic imaging could (Fig 3):

- Refer the patient to an emergency department (ED) or acute medical unit (AMU) for urgent assessment,
- Refer the patient to a hospital-based non-acute service (public outpatient department, private consultant review, or certain publicly available imaging modalities like X-rays/DEXA),
- Manage the patient in the GP setting, without access to the desired imaging.

By contrast, the new publicly funded pathway provides GP-directed access to private imaging, regardless of a patient’s insurance status. In this study, “managed in GP” refers to cases where the patient, after undergoing imaging, was not referred to a hospital for further evaluation.

### Free text analysis

Free-text comments from GPs were thematically analysed by two independent reviewers. First, the reviewers familiarized themselves with the comments, then generated initial codes through iterative discussion. Codes were subsequently organized into recurring themes, and the frequency of each theme was quantified to identify the most common perspectives. Data were managed using Microsoft Excel (Microsoft Corp., Redmond, WA) and R.

## Results

### Study overview and participant demographics

A total of 620 diagnostic imaging referrals were examined, originating from 45 GP clinics and involving 574 patients between April 2021 and September 2022. The initiative encompassed all nine CHO regions in Ireland. Patient ages ranged from 18 to 91 years, with a median age of 55. MRI comprised 65% of all referrals, most commonly of the spine (28% of tests), along with lower limb MRIs, DEXA scans, MRI brain studies, and chest X-rays.

### Impact on referral patterns

General practitioners provided two key pieces of information for each referral: how they would have managed the patient *before* the initiative (hypothetically) and how they *actually* managed the case under the new pathway. Comparisons must therefore be interpreted in light of potential recall bias, as the “pre-initiative” data are based on retrospective GP estimates rather than observed behaviour.

A statistically significant change in perceived referral patterns emerged (Exact McNemar test, p < 0.001). Most notably, for cases in which timely imaging was deemed necessary, referrals EDs or AMUs decreased by 89% (from 73 to 8). Public outpatient clinic referrals declined by 53% (from 281 to 132).

When standardized to a rate per 100 cases, ED/AMU referrals fell from 12 per 100 cases to 2 per 100, whereas outpatient referrals dropped from 45 per 100 to 21 per 100. Conversely, cases managed entirely in GP settings rose from 3 per 100 to 59 per 100. A full breakdown of numbers and percentages is provided in Table 1 (Appendix 2).”

### GP workload and patient care quality

In assessing the impact on GP workload, 35% of respondents reported an increase, 39% noted no change, and 26% observed a decrease. In terms of care quality, 91% of imaging studies were perceived to have improved patient care.

Thematic analysis of free-text comments (from 37 GPs) yielded four primary themes:

- **Reassurance/Reducing Anxiety** – Ninety-nine remarks highlighted the importance of imaging in easing worry, with subtopics including “Patient Reassurance” (66 mentions), “Patient Anxiety” (27), and “Care Team Reassurance” (6).
- **The Right Diagnosis** – Forty-nine comments addressed diagnostic clarity, featuring “Ruling Out Sinister Diagnosis” (36), “Significant Findings” (9), and “Incidental Findings” (4).
- **Quicker Care Closer to Home** – Seventy-seven instances described faster, local care, with 43 hospital referrals avoided and 32 advanced workups facilitated directly in the community.
- **Usage Beyond Intent** – Nine comments noted situations in which imaging was requested at the behest of non-GP healthcare professionals (5), or when patients requested MRI specifically before other modalities were considered (4).

These findings illustrate the perceived benefits and challenges of GP-directed imaging, including improved diagnostic certainty and patient reassurance, tempered by occasional increased workload and potential overuse.

## Discussion

### Summary

This study examined the perceived impact of a new HSE referral pathway in Ireland—GP Diagnostics—which enables GPs to order diagnostic imaging via private providers. GPs reported a notable decrease in hospital referrals, especially to EDs and AMUs, and an increase in cases managed directly in primary care. These findings align with Irish health policy goals focused on enhancing primary care capacity and reducing secondary-care waitlists, thereby expediting access to essential diagnostics and enabling more care to be delivered closer to home. However, the results should be interpreted with caution, given that they rely on retrospective GP assessments of how patients *might* have been managed in the absence of the initiative.

A substantial proportion of GPs perceived an increase in workload, yet 91% noted improved patient care when imaging was readily available. Thematic analysis identified key benefits, including reassurance to patients, greater diagnostic accuracy, and more localized, expedited care. These perceptions suggest that direct access to diagnostic imaging may substantially influence clinical decision-making and service delivery in general practice.

### Strengths and limitations

A major strength of this study is its breadth, encompassing 620 diagnostic studies across 45 clinics in all CHO regions, thereby capturing diverse demographic and geographic contexts. Nevertheless, the retrospective design introduces potential recall bias; GPs’ estimates of their “pre-initiative” referral patterns are hypothetical rather than directly observed. Additionally, information on turnaround times—from scan requests to results—was not collected, limiting understanding of how this interval might influence real-world decision-making. Hence, while the data offer valuable insights, they primarily reflect GP perceptions rather than definitive proof of altered referral behaviour.

### Comparison with existing literature

These findings support existing literature emphasizing the critical role of robust primary care in improving health system efficiency, consistent with Starfield’s work and the Irish Sláintecare vision [1, 2]. They also echo earlier Irish studies demonstrating that expanded access to diagnostics can reduce hospital referrals and admissions [5–8]. A persistent challenge, however, is the scarcity of comprehensive national data on diagnostic imaging usage and waiting times in Ireland. This research begins to fill that gap by illustrating how targeted interventions in primary care may yield meaningful shifts in care pathways.

### Implications for research and practice

Although this study relies on retrospective GP assessments, the findings suggest that improving GP access to diagnostic imaging may decentralize patient management away from hospitals, thereby alleviating pressure on secondary care. These observations align with Irish health policy objectives and could inform other health systems aiming to enhance primary care capacity. Nonetheless, robust investment in primary care resources and infrastructure is critical to sustain and expand this model.

Future studies, ideally incorporating objective referral data and longitudinal patient outcomes, are needed to confirm these initial insights and fully evaluate the initiative’s effectiveness. Although we did not collect data on socioeconomic, future research should examine whether GP Diagnostics reduces historic inequities in access among deprived populations.

Beyond patient and health system outcomes, future evaluations of GP Diagnostics could also consider potential planetary health impacts, such as reductions in patient travel to distant facilities and the patient perspective could also be evaluated in future research.

### Final remarks

From the GP perspective, the new GP Diagnostics referral pathway appears to enhance patient care quality and reduce reliance on hospital services, aligning with efforts to reinforce primary care as the cornerstone of the Irish health system. To build on these preliminary insights, ongoing monitoring of referral patterns and rigorous evaluation of patient outcomes are essential. We recommend continued refinement and expansion of this initiative, coupled with systematic data collection, to support evidence-based policy decisions and optimize healthcare delivery at both primary and secondary care levels.

## Data Availability

The data that support the findings of this study are not publicly available due to data protection restrictions.

## Appendix 1. Data Collection Format: GPs were asked to provide the following additional information for each care encounter:

Q1 If you didn’t have access to the HSE 'GP Direct Access to Diagnostic Imaging' initiative on the day what would you have done?

- Referred to Emergency Dept
- Referred to Acute Medical Unit
- Referred to Public Radiology Dept
- Referred to Public OPD
- Referred to Private Consultant
- Managed in GP
- Other (please specify) [__free_text__]

Q2 What actually happened as a consequence of the diagnostic imaging report?

- Referred to Emergency Dept
- Referred to Acute Medical Unit
- Referred to Public OPD
- Referred to Private Consultant
- Managed in GP
- Other (please specify- >) [__free_text__]

Q3 Did access to diagnostic imaging alter care for this patient?

- Yes: positive impact
- Yes: negative impact
- No
- Unsure

Q4 How has access to diagnostic imaging impacted your practice’s workload in the care of this patient?

- Significant increase in workload
- Some increase in workload
- No change
- Some decrease in workload
- Significant decrease in workload

Q5 “Are there any additional comments you’d like to share on the use of diagnostic imaging relevant to this patient’s case (optional)?”

- [__free_text__]

## Appendix 2.

**Table 1:** Referral destinations before and after the introduction of GP Diagnostics, as reported by GPs

	**Pre- GP Diagnostics**		**Post- GP Diagnostics**			
	**n**	**%**	**n**	**%**	**Absolute change**	**Relative change (%)**
Referrals to ED	44	7%	6	1%	- 38	-86%
Referrals to AMU	29	5%	2	0%	- 27	-93%
**Partial Sum** **Acute Secondary:** **ED/AMU**	**73**	**12%**	**8**	**1%**	**- 65**	**-89%**
Referrals to public outpatients	281	45%	131	21%	- 150	-53%
Referrals to private outpatients	61	10%	68	11%	+ 7	11%
Referrals to public radiology dept	157	25%	0	0%	- 157	-100%
**Partial Sum** **Nonacute Secondary:** **OP & Radiology**	**499**	**80%**	**199**	**32%**	**-151**	-54%
Managed in GP setting	16	3%	366	59%	+ 350	2188%
Other referral destinations	32	5%	47	8%	+ 15	47%

Pre-pathway figures represent retrospective GP estimates of how patients would have been managed in the absence of GP Diagnostics, while post-pathway figures reflect actual management decisions with GP Diagnostics access. Percentages are calculated relative to the total number of cases (n = 620). Absolute and relative change are based on counts. “Other referral destinations” include referrals outside of the categories specified (e.g., physiotherapist).
